# Editorial: What Are (Un)Acceptability and (Un)Grammaticality? How Do They Relate to One Another and to Interpretation?

**DOI:** 10.3389/fpsyg.2020.621267

**Published:** 2020-12-03

**Authors:** Susagna Tubau, Urtzi Etxeberria, Viviane Déprez, M.Teresa Espinal

**Affiliations:** ^1^Departament de Filologia Anglesa i de Germanística, Universitat Autònoma de Barcelona, Barcelona, Spain; ^2^Centre National de la Recherche Scientifique, IKER (UMR5478), Bayonne, France; ^3^Department of Linguistics, Rutgers University, New Brunswick, NJ, United States; ^4^Centre National de la Recherche Scientifique, ISC Marc Jeannerod (UMR5304), Bron, France; ^5^Departament de Filologia Catalana, Universitat Autònoma de Barcelona, Barcelona, Spain

**Keywords:** (un)acceptability, (un)grammaticality, interpretation, linguistics, experimental investigation

That grammatical sentences and their interpretation form the building blocks of linguistic theories is not controversial. Yet, the collection of articles in the present Research Topic shows that the notion of (un)grammaticality, on the one hand, and the observations of (un)acceptability ratings, on the other, can entertain in fact rather complex interactions. That is, the relation between the notion of grammaticality and the actual acceptability that speakers attribute to sentences is far from being straightforward: not only can some grammatical sentences present parsing difficulties that cause speakers to judge them unacceptable, but also sentences that are considered ungrammatical by linguists could be perceived as acceptable by speakers and lead to reliable interpretations. In addition, the methodology used in the investigation of (un)acceptability and (un)grammaticality and their relation may play an important role in our ultimate understanding of these two core notions which, despite being in principle independent from one another, often crisscross. Therefore, it seems useful and perhaps necessary to engage in actively evaluating how certain research methods can prove particularly useful when trying to establish the degree and extent to which (un)grammatical linguistic structures and their interpretations are (un)acceptable to speakers, and how this can be taken to reliably and consistently relate to (un)grammaticality.

As discussed in the Hypothesis and Theory article by Leivada and Westergaard, the relation between grammaticality, acceptability (and parsability), as found in the literature, is in need of terminological clarification, as (un)grammaticality and (un)acceptability do not homogeneously manifest coincident scales. Actually, further empirical confirmation that ungrammaticality can correspond to a speaker's misperception is found in the Original Research article by de-Dios-Flores where so called negative polarity item illusions in English are investigated. The author shows that grammatical sentences with multiple negations can be perceived as unacceptable under certain processing conditions. This complements previous research showing that ungrammatical sentences could be perceived as acceptable.

In a similar vein, the Original Research article by Blanchette and Lukyanenko offers empirical support to the idea that acceptability and grammaticality are not necessarily equated, thus making it possible for the grammar of English speakers to generate Negative Concord structures that are nonetheless judged with low acceptability ratings due to contextual factors. The Original Research article by Hubers et al. also illustrates that speakers of a language can use linguistic constructions that violate prescriptive rules. In their article they empirically investigate how one particular instance of these grammatical norm violations (i.e., the use of the equative particle instead of the comparative particle) is processed in Dutch and German. Results of three different experiments show that they are processed differently both from ungrammatical and grammatical sentences.

Also closely connected to acceptability is the frequency of occurrence in the context of language variation, as shown in the Original Research article by Gerasimova and Lyutikova. The authors address how different grammatical variants available to a single speaker in Russian distribute in production and perception, the main finding being that the more frequent a variant is, the higher the acceptability score speakers attribute to it. Nevertheless, the variants that are perceived as highly acceptable by the speakers are not always the ones that occur more frequently in production.

That methodological issues are relevant to the definition of acceptability and grammaticality is shown in the Original Research article by Langsford et al., who manipulate the instructions given to the participants of an experiment consisting in evaluating the acceptability/grammaticality of stimuli sentences to investigate whether instructions can help control variability in the motivation underlying ratings that has been identified in the literature. Their results show that participants indeed rate the sentences differently depending on whether they are asked to consider their acceptability or their grammaticality (the latter judgements being more extreme than the former).

The Brief Research Report by Gavarró shows that it is possible to use grammaticality judgement tasks with children, predicting that the differences in production and comprehension that children may display in comparison to adults will also show in a grammaticality judgement task, as production, comprehension, and grammaticality judgements would align. The author uses this methodology to investigate Relativized Minimality in child Catalan and argues that it can help determine whether it constitutes a grammatical or a processing phenomenon. The grammaticality judgement task is also the methodology used in Perpiñán's Original Research article on the sensitivity of L1 and L2 speakers of Spanish to extraction from island configurations. Perpiñán's experimental results show that L2 learners and native speakers use the same processing and interpretative mechanisms for parsing islands and point at the need to redefine grammaticality more holistically, as factors such as plausibility and processability might have a strong influence on it.

Oseki and Marantz use an acceptability judgement experiment to investigate morphologically complex words and evaluate five different computational models of morphological competence. Their results show that models with morpheme units outperform models without them. On the basis of the computational modeling of acceptability data, the authors show that morphological competence is best characterized as involving grammar-generated hierarchical structures rather than external surface linear strings in corpora.

On a related note, Huang and Ferreira discuss acceptability judgements in a Methods article. Acceptability judgements have been widely used in linguistic research, but have proven controversial, as they have a number of limitations and can include bias in the speakers' responses. This leads the authors to propose the application of Signal Detection Theory—a method used in other psychological research areas—to judgement data, with the aim of more effectively controlling bias. Further support that acceptability judgement methodology can blur conclusions on (un)acceptability is found in Wellwood's Hypothesis and Theory article. By considering a case study in degree semantics (i.e., adjective scale structure), the author proposes a two-step model of semantic interpretation that separates meaning from interpretation, and that relates language to thought.

In short, the articles in the present Research Topic confirm that it is indeed necessary to try to theoretically and empirically explore and (re)define (un)acceptability and (un)grammaticality as core notions that interact in complex ways, not only with one another, but also with the interpretation of sentences. This can certainly result not only in a better understanding of what makes (un)grammatical sentences (un)acceptable, but also of the role of performance factors, memory limitations, and processing mechanisms in the evaluation of (un)acceptability, (un)grammaticality, and the interpretation of linguistic structures. The methodological choices made when researching linguistic phenomena related to (un)acceptability, (un)grammaticality and/or their interaction have also been discussed as an essential piece of the research plan that should not be overlooked.

## Author Contributions

ST drafted the work, which was revised critically by UE, VD, and MTE. All authors contributed to the final approval of the work for publication.

## Conflict of Interest

The authors declare that the research was conducted in the absence of any commercial or financial relationships that could be construed as a potential conflict of interest.

